# A High Risk of Missing Congenital Cytomegalovirus-Associated Hearing Loss through Newborn Hearing Screening in Japan

**DOI:** 10.3390/jcm10215056

**Published:** 2021-10-29

**Authors:** Shujiro Bando Minami, Yoshiharu Yamanobe, Atsuko Nakano, Hirokazu Sakamoto, Sawako Masuda, Tetsuya Takiguchi, Sayaka Katsunuma, Tomoko Sugiuchi, Noriko Morita, Kimitaka Kaga, Tatsuo Matsunaga

**Affiliations:** 1 Department of Otolaryngology, National Hospital Organization Tokyo Medical Center, 2-5-1 Higashigaoka, Meguro, Tokyo 152-8902, Japan; 2 Division of Hearing and Balance Research, National Institute of Sensory Organs, National Hospital Organization Tokyo Medical Center, 2-5-1 Higashigaoka, Meguro, Tokyo 152-8902, Japan; kaga@kankakuki.go.jp; 3 Department of Otolaryngology, Head and Neck Surgery, School of Medicine, Keio University, 35 Shinanomachi, Shinjuku-ku, Tokyo 160-8582, Japan; nobe8756@gmail.com; 4 Division of Otolaryngology, Chiba Children’s Hospital, 579-1 Hetacho, Midori-ku, Chiba-shi 266-0007, Chiba, Japan; a.nkn@pref.chiba.lg.jp; 5 Department of Otolaryngology, Graduate School of Medicine Osaka City University, 1-4-3, Asahimachi, Abeno-ku, Osaka-shi 545-8585, Osaka, Japan; hsakamoto38@icloud.com; 6 Department of Otorhinolaryngology, National Hospital Organization Mie National Hospital, 357 Osato-Kubota, Tsu 514-0125, Mie, Japan; sawako3387@gmail.com; 7 Department of Otolaryngology, National Hospital Organization Kanazawa Medical Center, 1-1 Shimoishibiki-machi, Kanazawa-shi 920-8650, Ishikawa, Japan; ttakiguchi@me.com; 8 Department of Otolaryngology, Hyogo Prefectural Kobe Children’s Hospital, 1-6-7 Minatojima Minami-cho, Chuo-ku, Kobe-shi 650-0047, Hyogo, Japan; saya101477@gmail.com; 9 Department of Otorhinolaryngology, Kanto Rosai Hospital, 1-1 Kizukisumiyoshi-cho, Nakahara-ku, Kawasaki-shi 211-8510, Kanagawa, Japan; j.sugiuchi@cap.ocn.ne.jp; 10 Department of Otorhinolaryngology, Teikyo University Hospital, 2-11-1 Kaga, Itabashi-ku, Tokyo 173-8606, Japan; mori-ta@ninus.ocn.ne.jp; 11 Medical Genetics Center, National Hospital Organization Tokyo Medical Center, 2-5-1 Higashigaoka, Meguro, Tokyo 152-8902, Japan

**Keywords:** cytomegalovirus, newborn hearing screening, umbilical cord

## Abstract

It remains unclear to what extent newborn hearing screening (NHS) detects congenital cytomegalovirus (cCMV)-associated sensorineural hearing loss (SNHL) in Japan. This study aimed to clarify the NHS results and audiological characteristics of patients with cCMV-associated SNHL. A total of 541 individuals with unilateral or bilateral hearing loss of unknown etiology were examined for cCMV infection. cCMV infection was defined by the presence of CMV DNA in the dried umbilical cord detected using real-time quantitative PCR. NHS results and audiological data were retrospectively obtained from medical records. Forty-four cases (8.1%) were positive for cCMV infection. Of them, 33 cases underwent NHS and 13 cases (39.4%) passed NHS bilaterally. The pure-tone audiograms of 21 patients were obtained. There were seven cases of unilateral SNHL, five cases of asymmetric bilateral SNHL, and nine cases of symmetric bilateral SNHL. cCMV-related hearing loss is highly heterogeneous, and there is a high risk of missing this condition through NHS.

## 1. Introduction

Hearing loss due to congenital cytomegalovirus (cCMV) infection was first reported in 1964 [1]. cCMV infection is caused by vertical transmission from pregnant women via a primary cytomegalovirus (CMV) infection, reinfection, or reactivation. Symptoms range from asymptomatic to fatal and can cause permanent damage to the central nervous system and sensory organs [2]. Among them, sensorineural hearing loss (SNHL) is an important complication and is often progressively exacerbated. In a systematic review, the incidence of hearing loss in children with cCMV infection was 12.6%, and the rate of hearing loss due to cCMV infection in the population is estimated to be 0.5 per 1000 children [3]. cCMV is the most common cause of non-hereditary congenital SNHL and is estimated to be responsible for approximately 20–25% of SNHL cases among young children [4].

Infants are often infected with CMV via birth canal secretions, breast milk, their siblings, and the surrounding environment immediately after birth; consequently, CMV infection diagnosed using specimens collected after 21 days of age cannot be identified as congenital. Therefore, the detection of CMV DNA by isothermal nucleic acid amplification using urine within 21 days after birth is the gold standard for diagnosis of cCMV infection and has been covered by insurance policies since 2018 in Japan [5]. Dried blood spots (DBSs) taken within 1 week after birth for neonatal mass screening have been used to retrospectively study cCMV infection suspected after 21 days of age [6], although the sensitivity of CMV DNA detection in these specimens is low [7]. While these specimens are discarded after 1 year in Japan, the umbilical cord is kept at home as a symbol of the mother–child bond, and the sensitivity of CMV DNA detection is higher with umbilical cords than with DBSs [8]. The feasibility of using dried umbilical cords for retrospective diagnosis of cCMV infection was first reported in 2004 [9]. Molecular detection of CMV in preserved dried umbilical cord samples has been conducted in many studies of cCMV infection in Japan [10,11,12,13,14]. Reyes et al. also concluded that dried umbilical cords could play a complementary diagnostic role by comparing CMV viral load values between different samples [15].

Early hearing detection and early intervention have positively impacted the outcomes of children who are deaf or hard of hearing and their families across the world. Universal newborn hearing screening (NHS) has significantly lowered the average age of identification [16]. NHS is a necessary first step for children who are deaf or hard of hearing to promote language development. It remains unclear to what extent NHS detects cCMV-related SNHL in Japan. In this study, we investigated the medical records of SNHL patients with cCMV infection identified by PCR using their dried umbilical cords and clarified their NHS results and audiological characteristics.

## 2. Materials and Methods

This study was approved by the ethical committee of the National Hospital Organization Tokyo Medical Center and by the ethical committees of all research institutes who collaborated in this study. Informed consent was obtained by each research center from every participant or their parents.

From March 2010 to May 2019, 541 individuals with unilateral or bilateral hearing loss of unknown etiology were examined for cCMV infection. The inclusion criteria are those patients who (1) have unilateral or bilateral deafness with the degree of mild, moderate, severe, or profound; (2) have preserved umbilical cords and consented to the use of a part of them for CMV examination; and (3) developed hearing loss before the age of 20. The exclusion criteria are those patients who (1) are diagnosed with hearing loss due to environmental factors (meningitis, ototoxic drug, long-term noise exposure, etc.) other than cCMV infection, (2) have been clinically diagnosed as a syndromic hearing loss, or (3) are diagnosed with sudden deafness. cCMV infection was defined by the presence of CMV DNA in the dried umbilical cord detected using real-time quantitative PCR (SRL, Tokyo, Japan). Hearing levels of the patients were measured by pure-tone audiometry. NHS results were obtained from medical records or by interviewing the patients’ parents. NHS was performed using automated otoacoustic emission (AOAE) or automated auditory brainstem response audiometry (AABR) in Japan.

## 3. Results

### 3.1. cCMV Patients’ Characteristics

**Figure 1:**
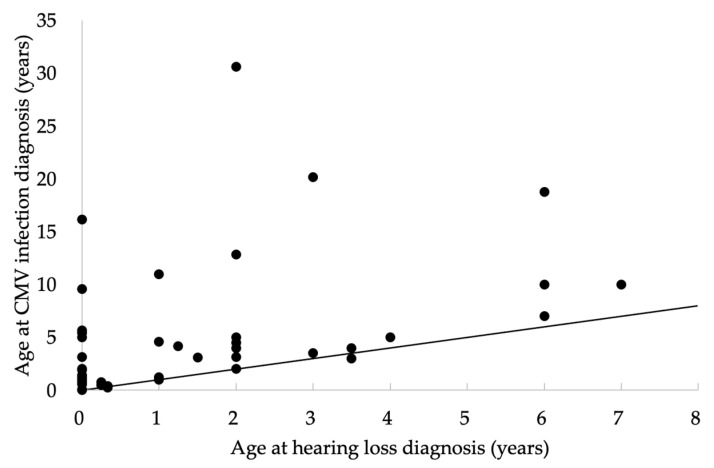
Relationship between age at hearing loss diagnosis and age at cytomegalovirus (CMV) infection diagnosis. The line indicates equal ages at hearing loss diagnosis and CMV infection diagnosis.

In total, 541 patients with unilateral or bilateral hearing loss with no apparent causes underwent umbilical cord CMV testing, and 44 (8.1%) were positive for cCMV infection. Of these 44 cases, 27 (61%) were female and 17 (39%) were male. Although the median age of SNHL diagnosis was 2 years (0–7 years), the median age of cCMV infection diagnosis was 3.5 years (0–30 years) (Figure 1). About 70% of all cases of CMV infection were diagnosed after 2 years of age.

### 3.2. NHS Survey of Children with Hearing Loss due to cCMV Infection

**Figure 2:**
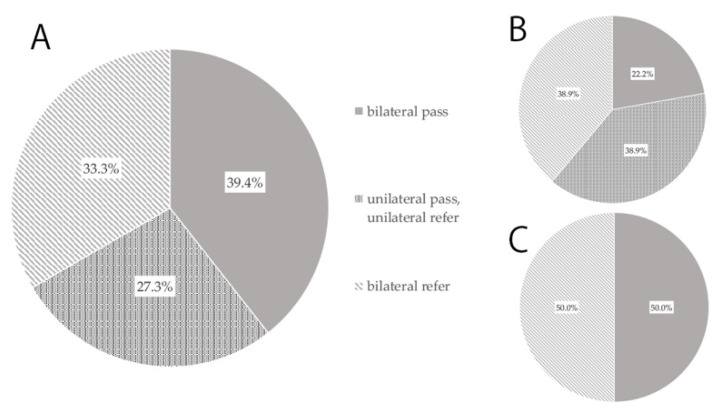
NHS survey of children with hearing loss due to cCMV infection. (**A**): Results of the 33 cases who underwent NHS. (**B**): Results of the 18 cases who underwent NHS by AABR. (**C**): Results of the six cases who underwent NHS by AOAE. The numbers indicate the percentage and the number of patients in parentheses.

We were able to determine whether 38 of the 44 cases underwent NHS. Thirty-three of them underwent NHS and five did not. Among these 33 cases, NHS was performed by AOAE in six cases, by AABR in 18 cases, and by unknown methods in nine cases. Thirteen cases (39.4%) passed bilaterally, eleven cases (33.3%) did not pass bilaterally, and nine cases (27.3%) passed unilaterally (Figure 2A). For NHS performed by AABR, four cases (22.2%) passed bilaterally, seven cases (38.9%) did not pass bilaterally, and seven cases (38.9%) passed unilaterally (Figure 2B). For NHS performed by AOAE, three cases (50.0%) passed bilaterally, and three cases (50.0%) passed unilaterally (Figure 2C).

### 3.3. Audiograms of Patients Who Were Diagnosed with Hearing Loss due to cCMV Infection

**Figure 3:**
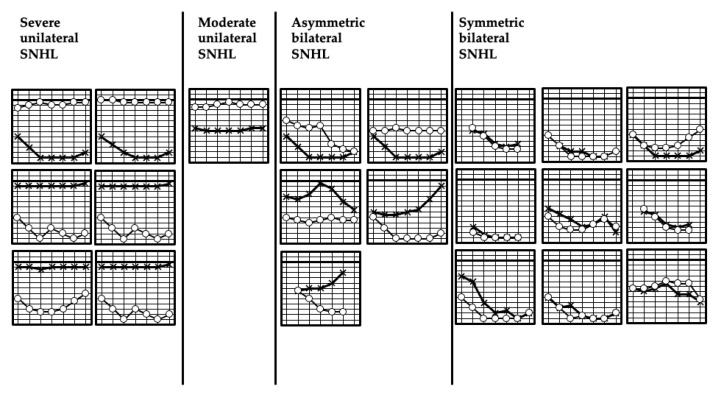
Audiograms of 21 patients who were diagnosed with hearing loss due to cCMV infection. SNHL, sensorineural hearing loss. P, U, and R indicate the NHS result of each case. P: bilateral pass, U: Unilateral pass and unilateral refer, R: bilateral refer.

Figure 3 shows the audiograms of 21 patients who underwent pure-tone audiometry. Six cases had severe unilateral SNHL, one case had moderate unilateral SNHL, five cases had asymmetric bilateral SNHL, and nine cases had symmetric bilateral SNHL.

## 4. Discussion

This study indicates that cCMV-related hearing loss is highly heterogeneous and there is a high risk of missing this condition through NHS. cCMV-related hearing loss is characterized by a high frequency of asymmetric hearing and the frequent occurrence of late-onset or progressive hearing loss.

Although the median age of SNHL diagnosis was 2 years (0–7 years) in this study, about 70% of all cases of CMV infection were diagnosed after 2 years of age. In contrast with hereditary SNHL, hearing loss due to cCMV infection is often asymmetric, which is a clue for diagnosis. Detection of CMV DNA using urine within 21 days after birth is the gold standard for diagnosis of cCMV infection and has been covered by insurance policies since January 2018 in Japan. cCMV infection is treated with valganciclovir [17] or ganciclovir [18] to improve hearing and prevent progression of hearing loss. Prospective randomized clinical trials indicate that these drug treatments are effective for children younger than 1 month. Evidence of the effectiveness of antiviral therapy is currently available only when treatment is started within 30 days after birth; therefore, it is necessary to definitively diagnose cCMV infection as early as possible. Iwasaki et al. reported that SNHL was detected in 4 (25%) of 16 infants with asymptomatic cCMV infection and that 2 (50%) infants who passed NHS had delayed-onset SNHL in follow-up examinations up to 4 years of age [19]. Gantt et al. reported that among 551 children with cCMV infection, 22 (4.0%) had hearing loss at birth and 71 (12.9%) subsequently developed hearing loss and passed NHS [20]. According to these previous reports, the percentage (39.4%) of cases who passed NHS in the current study may be an underestimation. AOAE is sensitive only to outer hair cell dysfunction, whereas AABR is sensitive to outer hair cell, inner hair cell, and auditory nerve dysfunction. Since both AOAE and AABR detected cCMV-associated hearing loss, it would be reasonable to assume inner ear dysfunction as the mechanism of the hearing loss. In addition, in this study, both AABR and AOAE missed cCMV-associated hearing loss, implying it is impossible to detect cCMV-associated delayed-onset hearing loss by either method. Current approaches to identify newborns with cCMV-related disease are inadequate, and most infants with cCMV infection will not receive timely and appropriate care in the absence of a screening program. Although immediate CMV screening is recommended for infants who do not pass NHS, such targeted cCMV screening will not capture infections that result in late-onset hearing loss. Universal screening offers larger net savings and the greatest opportunity to provide directed care. In the Tuscany region of Italy, screening for cCMV infection has been mandatory since 2008 for each newborn whose NHS result is “refer”, and 1.54% of all newborns screened for cCMV infection and 0.19% of infants submitted for NHS were positive for cCMV infection [21]. SNHL due to cCMV infection may fluctuate, progress, or be delayed; therefore, careful hearing monitoring should be continued. Infants diagnosed with cCMV infection require long-term follow-up, even if they pass NHS. The Joint Committee of Infant Hearing 2019 guidelines recommend that follow-up audiologic assessment of infants with cCMV infection is performed when they are no older than 3 months [16].

In this study, 67% of cases had bilateral SNHL and 33% of cases had unilateral SNHL, which is consistent with previous cohort studies. According to eight cohort studies [3], hearing loss due to cCMV infection was bilateral in 60% of cases and unilateral in 40% of cases (Table 1). A total of 55% of cases had bilateral severe-to-profound SNHL and cochlear implants should be considered in these cases, 25% of cases had late-onset SNHL, and 43% of cases had progressive SNHL. The incidence of SNHL due to cCMV infection depends on the timing of CMV infection during pregnancy. SNHL was detected in 80% and 8% of congenitally infected children who were infected after a primary maternal infection in the first and second trimester of pregnancy, respectively, and was not detected after primary maternal infection in the third trimester of pregnancy [22].

## 5. Conclusions

About 8% of individuals with unilateral or bilateral hearing loss of unknown etiology were positive for cCMV infection. cCMV-related hearing loss is highly heterogeneous and there is a high risk of missing this condition through NHS. Although immediate CMV screening is recommended for infants who do not pass NHS, such targeted cCMV screening will not capture infections that result in late-onset hearing loss. Universal cCMV screening offers larger net savings and the greatest opportunity to provide directed care.

## Figures and Tables

**Table 1:** The findings of eight cohort studies describing the characteristics of hearing loss due to cCMV infection. cCMV, congenital cytomegalovirus; NA, not available; SNHL, sensorineural hearing loss.

	Percentage of Cases with Bilateral SNHL	Percentage of Cases with Unilateral SNHL	Percentage of Cases with Bilateral Severe-to-Profound SNHL	Percentage of Cases with Delayed-Onset Hearing Loss	Percentage of Cases with Progressive SNHL
Saigal et al., 1982 [23]	57%	43%	43%	0%	0%
Preece et al., 1984 [24]	60%	40%	60%	0%	20%
Ahlfors et al., 1999 [25]	80%	20%	80%	0%	0%
Dahle et al., 2000 [26]	60%	40%	NA	31%	54%
Yamamoto et al., 2011 [27]	50%	50%	40%	NA	0%
Foulon et al., 2012 [28]	44%	56%	NA	13%	38%
Royackers et al., 2013 [29]	67%	33%	59%	11%	41%
Capretti et al., 2014 [30]	63%	38%	50%	63%	0%
Mean	60%	40%	55%	25%	43%
